# Giemsa-stained thick blood films as a source of DNA for *Plasmodium *species-specific real-time PCR

**DOI:** 10.1186/1475-2875-9-370

**Published:** 2010-12-22

**Authors:** Lieselotte Cnops, Marjan Van Esbroeck, Emmanuel Bottieau, Jan Jacobs

**Affiliations:** 1 Department of Clinical Sciences, Institute of Tropical Medicine (ITM), Antwerp, Belgium; 2 Faculty of Health, Medicine and Life Sciences (FHML), Maastricht, The Netherlands

## Abstract

**Background:**

This study describes the use of thick blood films (TBF) as specimens for DNA amplification with the *Plasmodium *species-specific real-time PCR that was recently validated on whole blood samples.

**Methods:**

The panel of 135 Giemsa-stained clinical TBFs represented single infections of the four *Plasmodium *species with varying parasite densities or only gametocytes, mixed infections, and negative samples and was stored for up to 12 years. Half of the Giemsa-stained TBF was scraped off by a sterile scalpel and collected into phosphate buffered saline. DNA was extracted with the Qiagen DNA mini kit with minor modifications. DNA was amplified with the 18S rRNA real-time PCR targeting the four *Plasmodium *species with four species-specific primers and probes in combination with one genus-specific reverse primer. Results of the PCR on TBF were compared to those of the PCR on whole blood and to microscopy.

**Results:**

Correct identification for single species infections was obtained for all TBF samples with *Plasmodium falciparum *(n = 50), *Plasmodium vivax *(n = 25), *Plasmodium ovale *(n = 25) and in all but one samples with *Plasmodium malariae *(n = 10). Compared to whole blood samples, higher Ct-values were observed by PCR on TBF with a mean difference of 5.93. Four out of five mixed infections were correctly identified with PCR on TBF. None of the negative samples (n = 20) gave a PCR signal. PCR on TBF showed a detection limit of 0.2 asexual parasites/μl compared to 0.02/μl for whole blood. Intra-run variation was higher for PCR on TBF (%CV 1.90) compared to PCR on whole blood (%CV 0.54). Compared to microscopy, PCR on TBF generated three more species identifications in samples containing a single species and detected the same four mixed-infections.

**Conclusions:**

Giemsa-stained TBFs are a reliable source of DNA for *Plasmodium *real-time PCR analysis, allowing applications in reference and research settings in case whole blood samples are not available.

## Background

Traditionally, microscopic examination of stained blood films remains the method of first choice for malaria diagnosis, both in endemic and non-endemic settings but also more recently developed molecular techniques have gained their place in malaria diagnosis, especially in reference centers [1-4]. Real-time PCR assays are particularly attractive because of the short turn-over-time and the avoidance of post-PCR contamination [5,6].

Although PCR is typically performed on whole blood samples [2,3,7], malaria diagnosis would benefit from the use of thick blood film (TBF) as a alternative source of DNA in case whole blood samples are not available. Indeed, stained blood films are frequently the only presented specimen for a second opinion in reference laboratories as whole blood samples require demanding storage and transport conditions. Moreover, it is known that species identification by microscopic examination might be difficult, and depends of the quality of the blood film [8,9]. In addition, archived blood film collections can be used for retrospective PCR analysis as demonstrated before [10-12].

The application of PCR on stored blood films was already demonstrated. Several reports indicated poor performance for low parasite densities [7,10,13] or interference of the staining [7,8,13-15]. Recently, a real-time PCR was developed and evaluated on whole blood samples that proved to be excellent in the detection of single and mixed infections [16] and showed a low detection limit; this incited us to apply this PCR for analysis of TBFs. The present study describes the successful use of Giemsa-stained TBFs for PCR detection and illustrates its use for malaria diagnosis in reference settings in case whole blood samples are not available.

## Methods

### Laboratory diagnosis of malaria at ITM

Clinical samples derived from patients suspected of malaria presenting at the outpatient clinic of the Institute of Tropical Medicine (ITM) Antwerp, Belgium or were submitted by Belgian laboratories to ITM for confirmation in the scope of the national reference function. Malaria diagnosis at ITM is accredited according to ISO 15189:2007 and done by the combination of standard microscopy, antigen detection and real-time PCR.

TBFs were made with approximately 20 μl venous blood, stained with Giemsa (pH 8.0) and examined by light microscopy using a × 500 magnification. Parasite density was expressed by the number of asexual parasites/μl. Species identification was done by microscopy on May-Grünwald Giemsa-stained thin blood films. After microscopic analysis, immersion oil was removed from the TBFs by xylene. After drying they were stored in a closed box at room temperature.

Antigen detection was done by the SD-FK60 Malaria Ag Pf/pan test (Standard Diagnostics, Hagal-Dong, Korea) for qualitative detection of *P. falciparum *histidine-rich protein-2 (HRP-2) and pan-species parasite lactate dehydrogenase (pLDH).

From January 2007, all samples that were positive with microscopy or antigen test were prospectively analyzed by real-time PCR on 200 μl of fresh EDTA-anticoagulated whole blood for confirmation or correction of the species identification. Whole blood samples of patients that were diagnosed between January 1997 and December 2006 were retrospectively confirmed or corrected by PCR on frozen samples as part of the validation of the recently developed real-time PCR [16]. The laboratory malaria diagnosis status was considered negative when microscopy, antigen detection and PCR were negative.

### Test samples

From the ITM collection of stored Giemsa-stained TBFs, a panel of malaria positive (n = 115) and malaria negative (n = 20) TBFs was selected. The selection was made so that the panel represented malaria positive TBFs with single infections of one of the four *Plasmodium *species (*Plasmodium falciparum, Plasmodium vivax, Plasmodium ovale, Plasmodium malariae*) with varying parasite densities (1 to 222,241/μl or only gametocytes) and different storage times (1 month to 12 years) and with mixed infections as determined by PCR on whole blood during laboratory malaria diagnosis (see above) (Table 1).

**Table 1:** Panel of TBF samples and corresponding parasite density used to evaluate the PCR on TBF.

	Laboratory malaria diagnosis						
		
Category^#^	*P. falciparum*	*P. vivax*	*P. ovale*	*P. malariae*	Mixed infection°	Negative	Total
0						20	20
0*	4	1					5
1-100	16	4	7	3			30
101-500	6	4	3	2	1		16
501-1000	6	3	5	1	1		16
> 1001	18	13	10	4	3		48

**Total**	50	25	25	10	5	20	135

# expressed as number of asexual parasites per microliter

* only gametocytes

° mixed infections consisted of Pf+Pm (n = 3), Pf+Po (n = 1) and Pf+Pv (n = 1)

In addition, two thin blood films of *P. falciparum *and *P. vivax *that were archived as teaching slides at the ITM for respectively 32 and 42 years were tested to reveal an idea about DNA recovery from very long-term stored blood films.

### Sampling and DNA extraction from TBFs

The TBF was split in two equal parts by tracing a straight and transversal line with a sterile scalpel (number 15, Farla Medicals, Antwerp, Belgium). Next, 10 μl of phosphate buffered saline (PBS; 0.02 M, pH 7.4) was dropped onto half of the surface of the TBF and this part was scraped off from the glass slide by making circular movements with the scalpel. The collected material was transferred in a sterile 1.5 ml tube that contained 90 μl PBS. DNA was extracted with the QIAamp DNA mini kit (Qiagen Benelux, Venlo, The Netherlands) according to the manufacturer's instructions but with reduced buffer volumes: only 100 μl of AL lysis buffer, 50 μl of ethanol and 50 μl of AE elution buffer was used. For each TBF a separate scalpel was used.

In addition, to evaluate alternative extraction methods and the influence of the Giemsa staining, unstained and Giemsa-stained TBFs and thin blood films of a *P. falciparum *sample with 60/μl were extracted according to 1) the Qiagen method described above, 2) the boiling method in Chelex-100 described by Kawamoto et al [17] and 3) the heating method in water described by Volpini *et al *[12].

### Real-time PCR on TBF

The 'four-primer' real-time PCR with a non-competitive design was used as described before [16]. Briefly, four *Plasmodium *species-specific forward primers and four *Plasmodium *species-specific probes together with one *Plasmodium *genus-specific reverse primer were used to target the 18S small subunit rRNA gene of the four *Plasmodium *species. Two duplex reactions, one to detect *P. falciparum *and *P. vivax *and another to detect *P. ovale *and *P. malariae*, were run in parallel for 2 min at 95°C followed by 50 cycles of 15 sec at 95°C and 60 sec at 60°C on the SmartCycler II (Cepheid Benelux, Bouwel, Belgium). Five microlitres of DNA was used in each reaction mixture.

Slides that were negative with the *Plasmodium *real-time PCR were analysed with a human beta-globin (HBB) real-time PCR to control for efficient DNA extraction and to rule out PCR inhibition. The HBB primers (200 μM) as described by Steinau *et al *[18] were used together with a Texas-Red labeled probe (400 μM) designed for real-time application (5'-TGCCCTCCCTGCTCCTGGGA-3'). The PCR was run for 15 min at 95°C followed by 50 cycles of 5 sec at 95°C, 20 sec at 60°C and 30 sec at 72°C on the SmartCycler II (Cepheid Benelux).

### Analytical sensitivity

To determine the analytical sensitivity, 10-fold serial dilutions were made from a single EDTA-blood sample infected with *P. falciparum *at a parasite density of 206,100/μl. A TBF was made of each dilution. PCR was performed on all TBF dilutions and on all dilutions of the whole blood. The highest dilution with a positive PCR signal indicated the detection limit.

### Reproducibility

To determine the reproducibility of the DNA extraction, eight TBFs were made from a single *P. vivax *sample and DNA was extracted. Comparison with the reproducibility on whole blood was done on eight fractions of another *P. vivax *sample with a comparable Ct-value. The subsequent PCR runs on TBF and whole blood were performed in the same run and the variation coefficient (%CV) of the Ct-values for TBF and whole blood were calculated.

### Data analysis

Improved DNA detection was determined by subtracting Cycle threshold (Ct)-values measured by the PCR on TBF and on whole blood and is indicated by ΔCt. Statistical differences between logarithmic Ct-values of both specimen types were determined by paired *t-test *analysis. The mean of all ΔCt-values was calculated together with the ± 95% confidence intervals (±CI 95%) of the mean ΔCt.

## Results

### Sampling and testing of DNA extraction method

From preliminary test assays (Table 2), it is clear that the Qiagen extraction method is superior to the Chelex and water boiling method for the recovery of DNA from blood films. Comparison of *P. falciparum *and HBB Ct-values obtained with the Qiagen method indicated that no large differences were observed for unstained versus stained TBFs and thin blood films. In contrast, no parasite or human DNA was detected in the samples extracted with the Chelex method. With the water heating method, only human DNA was detected with high Ct-values in unstained blood films. In relation to the lower blood volume applied onto thin blood films, slightly higher Ct-values were seen compared to TBFs. Based on these results, stained TBFs were used in all further assays as specimen type and they were processed by the Qiagen method.

**Table 2:** Comparison of three DNA extraction methods for optimal recovery of DNA from unstained and stained TBFs and thin blood films.

	QIAGEN method		Water method		Chelex method	
	
	Ct Pf	Ct HBB	Ct Pf	Ct HBB	Ct Pf	Ct HBB
unstained TBF	38,21	33,92	0	44,69	0	0
stained TBF	38,92	32,25	0	0	0	0
unstained thin blood film	39,43	35,32	0	40,19	0	0
stained thin blood film	39,90	34,80	0	0	0	0

Cycle threshold (Ct)-values were given for the detection of parasite DNA (Pf: *Plasmodium falciparum*) and human DNA by human beta globin (HBB) PCR.

### Panel of clinical samples

Table 1 lists the panel of 135 TBFs representing single infections of the four *Plasmodium *species (n = 110), mixed infections (n = 5), and negative samples (n = 20) and indicates the parasite density levels by categories ranging from 1-100 (n = 30), 101-500 (n = 16), 501-1,000 (n = 16) and more than 1,000 (n = 48) asexual parasites/μl. Five of the samples with single infections contained only gametocytes of *P. falciparum *(n = 4) or *P. vivax *(n = 1) (Table 1). The malaria positive TBFs (n = 115) were stored for one month to two years (n = 66), 3 to 5 years (n = 15), 6 to 8 years (n = 21) or 9 to 12 years (n = 13).

### Diagnostic sensitivity of PCR on TBF

Compared to PCR on whole blood as the reference method, PCR on TBFs identified 109 out of the 110 (99.1%) single *Plasmodium *infections: all samples containing *P. falciparum *(n = 50), *P. vivax *(n = 25) and *P. ovale *(n = 25) were correctly detected as well as all but one containing *P. malariae *(n = 10) (Table 3). In the latter sample, microscopic examination of only the thin blood film, and not of the TBF, revealed a single schizont and no other asexual parasites. The HBB PCR to control the extraction did not give a signal. Additional analysis on the other half of the TBF gave again a negative result for *P. malariae *but revealed a HBB Ct-value of 44.43. The TBF was stored for 1 year and 11 months.

**Table 3:** Species identification by real-time PCR on TBF and real-time PCR on whole blood as reference method.

	PCR on whole blood						
	
PCR on TBF	*P. falciparum*	*P. vivax*	*P. ovale*	*P. malariae*	Mixed infection	Negative	Total
*P. falciparum*	50				1		51
*P. vivax*		25					25
*P. ovale*			25				25
*P. malariae*				9			9
Mixed infection					4		4
Negative				1		20	21

**Total**	50	25	25	10	5	20	135

Compared to microscopy, PCR on TBF generated three additional species identifications (one *P. falciparum*, one *P. vivax *and one *P. ovale*) in which microscopy detected *Plasmodium *parasites without an unambiguous species identification (Table 4).

**Table 4:** Species identification by real-time PCR on TBF and microscopy as reference method.

	Microscopy							
		
PCR on TBF	*P. falciparum*	*P. vivax*	*P. ovale*	*P. malariae*	Mixed infection	*Plasmodium spp*.	Negative	Total
*P. falciparum*	51					1		51
*P. vivax*		24				1		25
*P. ovale*			24			1		25
*P. malariae*				9				9
Mixed infection					4			4
Negative				1			20	21

**Total**	51	24	24	10	4	3	20	135

Compared to PCR on whole blood, four of the five mixed-infections were correctly identified with PCR on TBF (one *P. vivax/P. malariae *co-infection and three *P. falciparum/P. malariae *co-infections) (Table 3). One *P. ovale *with a Ct-value of 37.48 when detected by PCR on whole blood was missed in a co-infection with *P. falciparum *and neither diagnosed with microscopy (Table 4).

### Diagnostic specificity of PCR on TBF

No *Plasmodium *DNA amplification was seen in all twenty TBFs from samples that were negative with standard microscopy, antigen detection and PCR on whole blood, indicating a diagnostic specificity of 100%. The HBB PCR revealed in all those samples a positive signal.

### Analytical sensitivity and reproducibility of PCR results

The detection limit was 0.2 asexual parasites/μl for PCR on TBF compared to 0.02 for PCR on whole blood. Reproducibility testing demonstrated a coefficient of variation of 1.90 for PCR on TBF while for PCR on whole blood a variation of only 0.54 was observed.

### Comparison of Ct-values of the PCR on TBF and on whole blood

The Ct-values of each sample measured by PCR on TBF and by PCR on whole blood were significantly different. The mean ΔCt-value was 5.93 (± 0.43) with a maximum difference of 12.57 Ct-values in a *P. ovale *sample with 480 parasites/μl that was stored for 8 years and 11 months and a minimum difference of 1.79 Ct-values in a *P. falciparum *sample with 32 parasites/μl that was stored for eight years and eight months. The difference in Ct-value between PCR on TBF and PCR on whole blood was consequently seen for all samples regardless the parasite density or storage time.

Additional PCR analysis demonstrated the ability to detect *P. falciparum *DNA with a Ct-value of 35.32 and *P. vivax *DNA with a Ct-value of 39.80 in the two thin blood films that were archived for respectively 32 and 42 years.

### Contamination

Of note is that during this entire study, a contamination was observed once during the TBF extraction procedure. One single infection by *P. ovale *was identified as a mixed infection with *P. falciparum *by PCR on TBF, probably because of cross-contamination by the preceding TBF containing *P. falciparum *that was extracted in the same batch. A second extraction on the other part of the TBF confirmed the single infection with *P. ovale*.

## Discussion

In this study, the ability of real-time PCR to amplify DNA extracted from Giemsa-stained TBFs was evaluated. Challenged with a panel of archived clinical TBF samples, the PCR proved accurate in the detection of single and mixed species infections on samples of the four *Plasmodium *species with varying parasite densities and stored for up to 12 years.

This study is the first to report real-time PCR analysis on TBFs allowing a fast turn-around time and a high diagnostic sensitivity. Previous studies used either conventional or nested PCR assays and focused on a single species, mostly *P. falciparum *[10,14,19-21]. Compared to PCR on whole blood, 99.1% single infections of the four *Plasmodium *species and four of the five mixed infections were detected which is excellent in comparison to previous studies that demonstrated a diagnostic sensitivity of 71% [20] or 85.6% [22]. Despite the low blood volume, Giemsa staining and months to years of storage, *Plasmodium *species identification was possible by PCR on TBFs.

Noteworthy is that also samples with low parasite densities were correctly identified. Earlier PCR studies on TBFs did not include [11,15] or were not able to detect low parasite density samples [7,20]. Detection of low parasite densities is of particular importance since rapid diagnostic tests, frequently used as an adjunct for the diagnosis of malaria, have low sensitivities at parasite densities below 100/μl and 500/μl for *P. falciparum *and the non-*falciparum *species respectively [23-27]. Likewise can the detection by PCR on TBF of samples with only gametocytes be considered as an advantage in the non-endemic settings, as patients may still be diagnosed after empiric (self)-treatment.

With PCR on TBFs, only one single infection with *P. malariae *was missed. This may be explained by sample error: PCR on the corresponding whole blood sample demonstrated the presence of *P. malariae *DNA and microscopic analysis of the thin blood film, but not of the TBF, revealed only one schizont of *P. malariae*.

One of the advantages of the *Plasmodium *real-time PCR on whole blood is its high analytical sensitivity [16]. Applied on TBF however, the PCR showed an analytical sensitivity which was 10 times lower. This might be explained by differences in sample volume (equivalent to ~10 μl for TBF versus 200 μl for whole blood). Despite this, the actual detection limit of 0.2 asexual parasites/μl is much lower than those previously described for other PCR assays on TBF reporting values of 20, 500 and 3,500/μl respectively [7,10,13]. The low sample volume of the TBF probably also explains the higher intra-run variation as compared to whole blood. The lower concentration of extracted DNA from TBFs is also reflected by the higher Ct-values as observed by PCR on TBF. The negative result of the *P. ovale *in the mixed infection could be explained by this as the amount of extracted DNA from the TBF of this minor species was probably below the limit of detection.

For sampling, half of the TBF surface was used, preserving the other half for additional microscopic or molecular examinations. DNA extraction was done by minor adaptation of the easy format of Qiagen mini-spin columns frequently used for whole blood samples. No influence of the staining was seen with this method. Indeed, column-based extraction methods include washing steps to remove unwanted inhibitory PCR factors. Some studies described the use of a simple boiling method in Chelex or water for DNA extraction [12,17,20,22] but preliminary results of those methods proved to be not successful in this study. This indicates that highly purified DNA is required for real-time PCR assays and the need to control efficient extraction in negative TBFs by human beta globine PCR. Attention should be paid during manipulations of scraping and collecting TBF material carrying the inherent risk of contamination as experienced once in this study. Likewise, storage and staining conditions, immersion oil and xylene have been described as sources of *Plasmodium *DNA contamination [15,20].

Despite the excellent performance of the PCR on TBF samples, whole blood samples are the first choice for malaria diagnosis by PCR and the use of TBF samples are only considered when whole blood is not available. This choice is argued by the higher accuracy of the PCR when applied on whole blood, and by the concerns of contamination, despite rigorous procedures.

A limitation of the present study is its retrospective design that did not allow for all samples side-by-side comparison of the Ct-value obtained from stored TBF and from fresh whole blood. However, it is of note that more than 40% of the TBF samples had been stored for more than three years and that PCR was successfully applied on TBF samples stored for up to 12 years. Li *et al *[11] demonstrated malaria RNA in blood smears stored up to 20 years and Volpini *et al *[12] detected *Leishmania *DNA in a slide that was stored up to 36 years. Noteworthy, *P. falciparum *and *P. vivax *DNA was successfully amplified from two stained teaching slides that were stored for respectively 32 years and 42 years.

Another limitation of the present study is that the real-time PCR assay does not detect *Plasmodium knowlesi*, a simian malaria parasite that can cause malaria in humans and is mainly distributed in Southeast-Asia [28,29]. Some studies reported the detection of *P. knowlesi *by PCR in returned travellers [30-32]. The well-defined panel of clinical samples used in this study probably does not contain this rare species. But awareness is needed for travellers returned from *P. knowlesi *endemic regions that demonstrate microscopy positive results with parasites that resemble *P. malariae *or *P. falciparum *morphology and that give PCR negative results. A *P. knowlesi*-specific probe and forward primer have been recently designed at the laboratory of ITM but the evaluation of the PCR multiplex design to detect this additional species is still under investigation.

What may be the applications of the current PCR on TBF? From the present results, it is clear that PCR on TBF is a reliable alternative in case whole blood is not available, and thus of added value for reference malaria diagnosis. Its detection limit is still below that of microscopy in reference settings, *i.e. *close to 10-50/μl [1,2,33] and that of malaria rapid diagnostic tests which can only be performed on whole blood samples. This means that PCR applied on TBF can confirm or rule out the diagnosis of malaria and can confirm or adjust species identifications made by microscopy alone. For instance, PCR can generate species identification for parasites that are notoriously difficult to distinguish from each other (like *P. ovale *and *P. vivax*) [34,35], and for samples with unambiguous species identification by microscopy because of low parasite densities, poor staining quality, altered parasite morphology due to treatment or for the detection of mixed species infections.

TBF samples have logistic advantages over whole blood. They are frequently stored for reasons of traceability, and they are less demanding for storage and shipment as compared to whole blood [36]. In that way, applications of PCR may also be foreseen in research settings: TBFs are part of standard laboratory work-up of patients suspected of malaria both in endemic and non-endemic settings and may be used for further work-up, thereby avoiding the need for extra sampling of blood. This may be important especially in vulnerable patients groups (such as children) and in communities who are reluctant to blood sampling [18,37]. In addition, TBFs can easily be sent to reference laboratories for quality control of field study results.

## Conclusions

In conclusion, the present study demonstrated that Giemsa-stained TBFs can be a reliable alternative source of DNA through a simple sampling and extraction protocol. Other real-time PCR assays or amplification methods might adapt and use this platform, offering opportunities of studying clinical samples and archived collection samples for epidemiological relationships and resistance markers.

## List of abbreviations

CI: Confidence interval; Ct: Cycle threshold; CV: Coefficient of variation; DNA: Desoxyribonucleotide acid; EDTA: Ethylene diamine tetra-acetic acid; HBB: Human beta-globin; HRP-2: Histidine-rich protein-2; ITM: Institute of Tropical Medicine; PBS: Phospate buffered saline; PCR: Polymerase chain reaction; pLDH: *Plasmodium*-specific parasite Lactate dehydrogenase; TBF: Thick blood film.

## Competing interests

The authors declare that they have no competing interests.

## Authors' contributions

LC and JJ designed the study protocol. MvE and EB organized and coordinated the sample collection. LC performed PCR analysis, analysed and interpreted the results. LC, MvE, and JJ drafted the manuscript. LC performed the statistical analysis. All authors read and approved the final manuscript.
